# Investigating the evolution process of lung adenocarcinoma via random walk and dynamic network analysis

**DOI:** 10.3389/fgene.2022.953801

**Published:** 2022-09-29

**Authors:** Bolin Chen, Jinlei Zhang, Teng Wang, Ci Shao, Lijun Miao, Shengli Zhang, Xuequn Shang

**Affiliations:** ^1^ School of Computer Science, Northwestern Polytechnical University, Xi’an, China; ^2^ School of Information Technology, Minzu Normal University of Xingyi, Xingyi, China

**Keywords:** evolution process, dynamic network, random walk, Monte Carlo, lung adenocarcinoma

## Abstract

Lung adenocarcinoma (LUAD) is a typical disease regarded as having multi-stage progression. However, many existing methods often ignore the critical differences among these stages, thereby limiting their effectiveness for discovering key biological molecules and biological functions as signals at each stage. In this study, we propose a method to discover the evolution between biological molecules and biological functions by investigating the multi-stage biological molecules of LUAD. The method is based on the random walk algorithm and the Monte Carlo method to generate clusters as the modules, which were used as subgraphs of the differentiated biological molecules network in each stage. The connection between modules of adjacent stages is based on the measurement of the Jaccard coefficient. The online gene set enrichment analysis tool (DAVID) was used to obtain biological functions corresponding to the individual important modules. The core evolution network was constructed by combining the aforementioned two networks. Since the networks here are all dynamic, we also propose a strategy to visualize the dynamic information together in one network. Eventually, 12 core modules and 11 core biological functions were found through such evolutionary analyses. Among the core biological functions that we obtained, six functions are related to the disease, the biological function of neutrophil chemotaxis is not directly associated with LUAD but can serve as a predictor, two functions may serve as a predictive signal, and two functions need to be verified through more biological evidence. Compared with two alternative design methods, the method proposed in this study performed more efficiently.

## 1 Introduction

Lung cancer is a type of malignant tumor with the highest morbidity and mortality in the world at present (Siegel et al., 2016; Siegel et al., 2020; WHO, 2020; Rahib et al., 2021), while lung adenocarcinoma (LUAD) has become the most important pathological type of lung cancer, accounting for the vast majority of all lung cancer patients. LUAD is a typical disease whose progression is a dynamic process that typically occurs from a normal state through the gradual accumulation of small changes in biological molecules that eventually result in a disease state. Therefore, it is of particular interest to analyze the multi-stage biological molecules of LUAD in order to help cure the disease.

Recent developments in experimental procedures have resulted in several mature methods to analyze biological molecules. One approach involves directly using gene set enrichment analysis (GSEA) to identify crucial biological molecules in LUAD (Murohashi et al., 2010; Subramanian et al., 2005; He et al., 2019) and age-related macular degeneration (AMD) (Zhang et al., 2014). Another approach involves clustering biological molecules into networks, using existing clustering algorithms, such as random walk (Liu et al., 2016; Zhang et al., 2019), Markov clustering (MCL) (Yang et al., 2019), clustering with overlapping neighborhood expansion (ClusterONE) (Nepusz et al., 2012), SigMod (Liu et al., 2017), and unsupervised hierarchical clustering (Sato et al., 2014), in order to clarify the significant biological molecules and related biological functions. However, the limitation of directly using GSEA analysis lies in the inability to classify biological molecules for correlation with and the subsequent refinement of a portion of biological molecules corresponding to certain specific biological functions. Although module generation via clustering algorithms is a good idea, there are problems with the details in relation to the network being considered. Hence, the existing methods are not ideal for discovering key biological molecules and biological functions as signals for this multi-stage disease.

To overcome this limitation, the present work aims to identify the crucial biological molecules of LUAD and the biological functions that accompany LUAD through its four stages, ultimately aiming to improve the diagnosis, treatment, and prognosis of LUAD. The aim is to discover the evolution between biological molecules and biological functions by investigating the multi-stage biological molecules of LUAD.

Many current methods for clustering biological molecules in order to identify key biological molecules use the random walk algorithm. However, differing from the traditional random walk algorithm, we combine the Monte Carlo method with a random walk approach, which does not record the start vertex and the end vertex of the walking path but instead assigns a weight to every step in the walking. Moreover, studies on networks that use the traditional random walk algorithm can provide only a generic solution, lacking in specificity. Thus, we consider the degree of the vertices to help define the weight by introducing the penalty coefficient.

**FIGURE 1:**
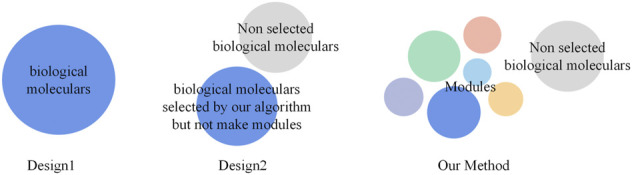
Schematic diagram of the three methods. Design1 presents the approach that directly gathers all the biological molecules in each stage in GSEA to obtain the results. Design2 presents the approach that gathers the biological molecules obtained through our clustering algorithm without dividing them into different modules in GSEA to obtain results. Our method uses a clustering algorithm to generate modules and put biological molecules in specific modules to obtain the results.

Specifically, the steps followed in the present work are as follows. The stage-specific differentiated biological molecule network was first built, which contains genes, miRNA, and lncRNA, respectively. An innovative clustering method based on the random walk (Liu et al., 2016) algorithm and the Monte Carlo method (Kroese et al., 2014) was proposed to cluster the biological molecules, and only appropriate clusters were selected as modules, which were believed to have strong relationships with specific biological functions. Subsequently, the modules were connected to generate the global module network for individual stages, and the Jaccard coefficient was employed to find relationships between modules from adjacent stages. GSEA was used to gather the biological functions into modules, and the obtained cellular functions were used to build a functional interaction network by taking individual functions as vertices and their Jaccard coefficient as edges. The combination of the aforementioned two networks into a comprehensive network can be used to explore the evolutionary relationships of biological functions according to the obtained modules. The key evolution information in the comprehensive network was selected to build the core evolution network. The key ideas of this proposed method are shown in Figure 1.

## 2 Methods

### 2.1 General framework

The dataset in the experiments comprises the gene, miRNA, and lncRNA information for patients with LUAD (encompassing the four stages). The datasets were downloaded from The Cancer Genome Atlas (TCGA) database (https://portal.gdc.cancer.gov/), including tumor and normal groups, and we obtained the data for mRNA, miRNA, and lncRNA. The genome annotation for *Homo sapiens* was downloaded from the Gencode database (https://www.gencodegenes.org/). Duplicate genes were removed and standardized using the trimmed mean of M-values (TMM) normalization (Robinson et al., 2010). Moreover, taking miRNA as the object, the correlation data verified by experiments were kept for further analysis. The final form of the datasets presents the data regarding edge sets of the four stages of the disease, where four kinds of edges were considered, namely genes and genes, miRNAs and lncRNAs, genes and miRNAs, and genes and lncRNAs.

**FIGURE 2:**
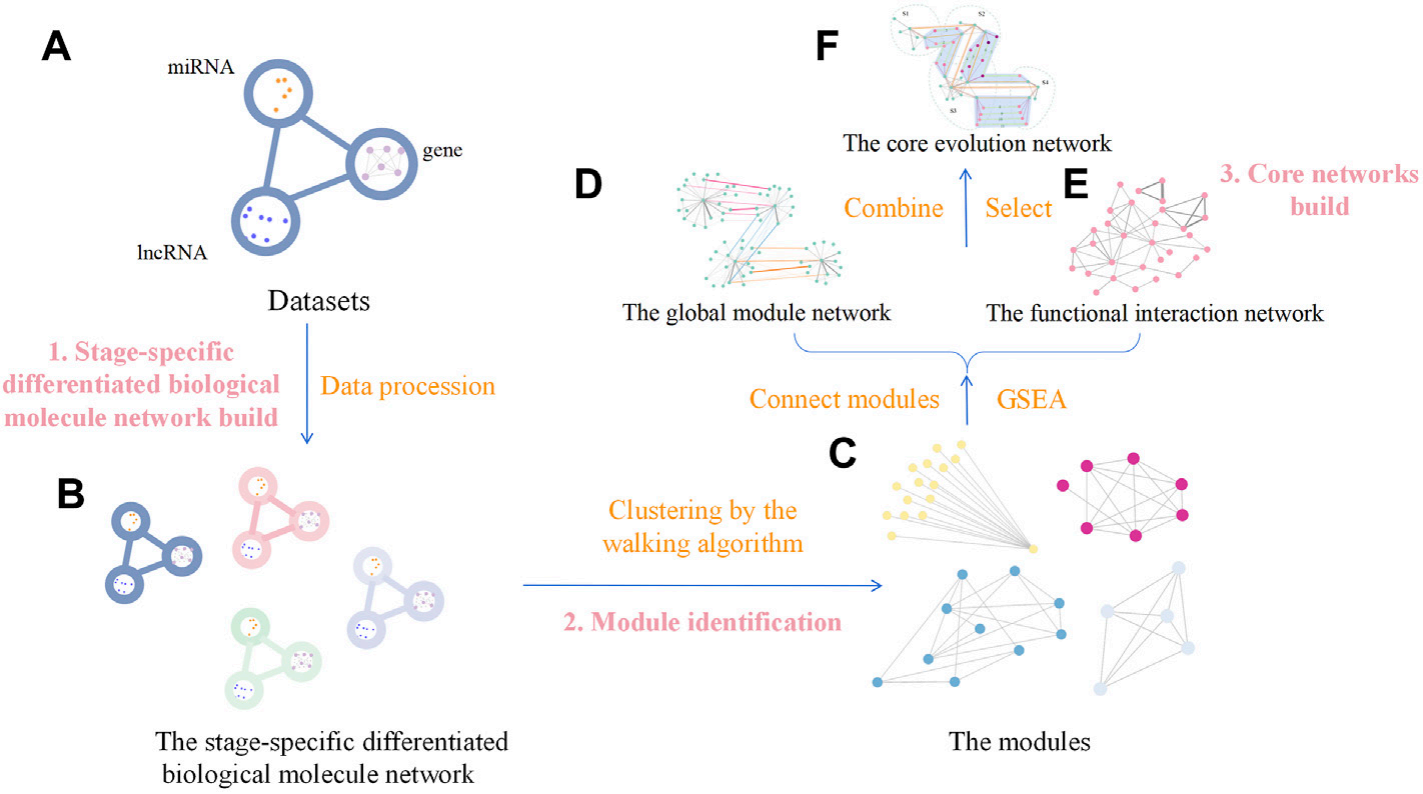
General framework of our method. It comprises three steps. Step (1) is building the stage-specific differentiated biological molecule network build, and the method used is data processing, which transforms the four datasets **(A)** into the stage-specific differentiated biological molecule network **(B)**. Step (2) is identifying the modules, which uses the walking algorithm to generate modules **(C)** from the network **(B)**. Step (3) is building the core networks, which initially connects modules to generate the global module network **(D)** and, through GSEA, to generate the functional interaction network **(E)** from the modules **(C)**. We then use the two networks **(D,E)** to generate the core evolution network **(F)** by combining and selecting.

The analysis process comprised three steps: 1) building the stage-specific differentiated biological molecule network, 2) module identification, and 3) building the core networks. In the first step (building the stage-specific differentiated biological molecule network), the dataset was transformed into the differentiated biological molecule network in each stage, including not only the differentiated genes but also the miRNA and lncRNA. In the module-identification step, modules of the differentiated biological molecule network were obtained by clustering the vertices and edges in the network through the innovative walking algorithm. The source code for the algorithm in this step is available on Github (https://github.com/Thunder-ZJL/InnovativeRandomWalk-MonteCarlo). The last step (building the core networks) concerned how to build the global module network by connecting modules in each stage, further using similarity to connect modules in adjacent stages, and how to build the functional interaction network using the Gene Ontology (GO) database and the Kyoto Encyclopedia of Genes and Genomes (KEGG) in GSEA to obtain the biological functions corresponding to the modules. This step also concerned how to build the core evolution network through the combination of the two aforementioned networks, as well as how to find the core biological molecule modules and the core biological functions. The general framework of the proposed method is shown in Figure 2.

### 2.2 Buidling the stage-specific differentiated biological molecule network

**FIGURE 3:**
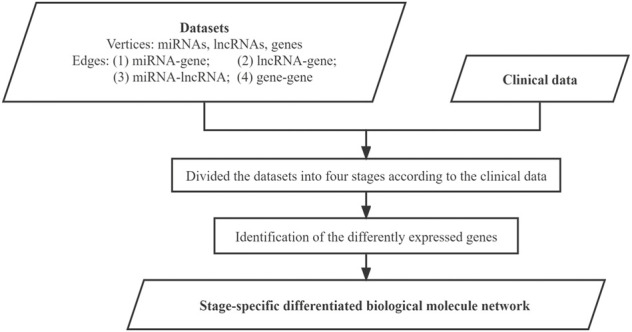
Flowchart for building the sage-specific differentiated biological molecule network.

The datasets were used to construct the differentiated biological molecule network for each stage, where the vertices represent the differently expressed genes, miRNAs, and lncRNAs, while the edges represent their functional interactions between these biological molecules. The integration of miRNAs and lncRNAs increases the degree of the meaningfulness of the identified molecule modules from individual networks. The specific work in this step is shown in Figure 3.

### 2.3 Module identification

Clustering refers to grouping vertices and edges with the same or similar functions in the network into corresponding clusters. In this step, we used a clustering algorithm to cluster the vertices in the stage-specific differentiated biological molecule network and to pick the key clusters as molecule modules. Molecule modules are dense subgraphs of the individual stage-specific biological molecule network. They are a response to specific biological functions that play important roles in the progression of the disease.

Random walk is a network clustering algorithm based on a very simple idea (Firat et al., 2007). Traditionally, mature methods based on a random walk algorithm are used to identify the key biological molecules and obtain biological functions through clustering; however, using a traditional random walk algorithm results in false positive or false negative results. Thus, considering that different networks have different vertex-edge characteristics, we considered the degree of the vertices to balance the weights so that the results do not tend to the vertices with a high degree.

**FIGURE 4:**
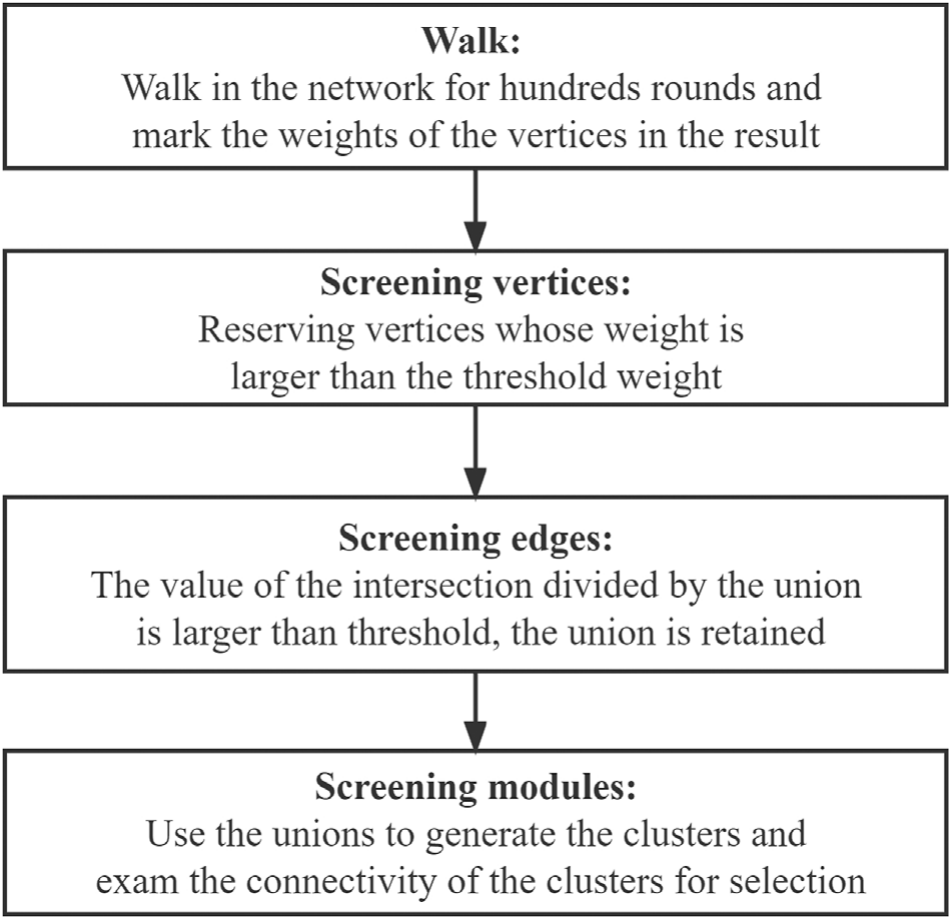
Flowchart for our algorithm.

In this step, a clustering algorithm based on the random walk algorithm together with the Monte Carlo method is proposed, and the penalty strategy was employed to further assess the quality of the obtained walks. Unlike the traditional random walk algorithm, where randomness is merely reflected in the start vertex and end vertex of the walk, our proposed algorithm focuses on every vertex in the walk, and every random choice is evaluated according to its frequency and importance. In other words, every step in the path of walking is recorded, which is more complete than only recording the starting point and the end point in the traditional random walk algorithm. The flowchart showing the design idea of our algorithm is presented in Figure 4.

Figure 4 shows the process for obtaining the connected subgraph, the module, screening the vertices in the network, screening the edges among the edges composed of the vertices, and screening the modules formed by the vertices and edges.

In the walk part and the vertices-screening step, we used the weight to represent the sequence of the order of appearance in the walk. More specifically, *N* was taken as the basic weight, in which the start vertex weights *N* and the end vertex weights 1. Furthermore, to avoid the final results tending to the vertices that have smaller degrees, we introduced the penalty coefficient to ensure balance. The design of the penalty coefficient aims to consider the degree of the vertices in the network.

Through walking, the weights of the vertices were determined, and the final weights were employed to generate a weight distribution along with the walk. The vertices whose weight was larger than a specific threshold were selected for further statistical analysis.

In the edges-screening step, both the intersection and the union between the pairwise vertices among all the selected vertices in the network were made. Based on the value of the set size of the intersection divided by the union, a threshold of [0,1] was selected as the screening condition, and any union whose value was larger than the threshold was retained for further statistical analysis.

**FIGURE 5:**
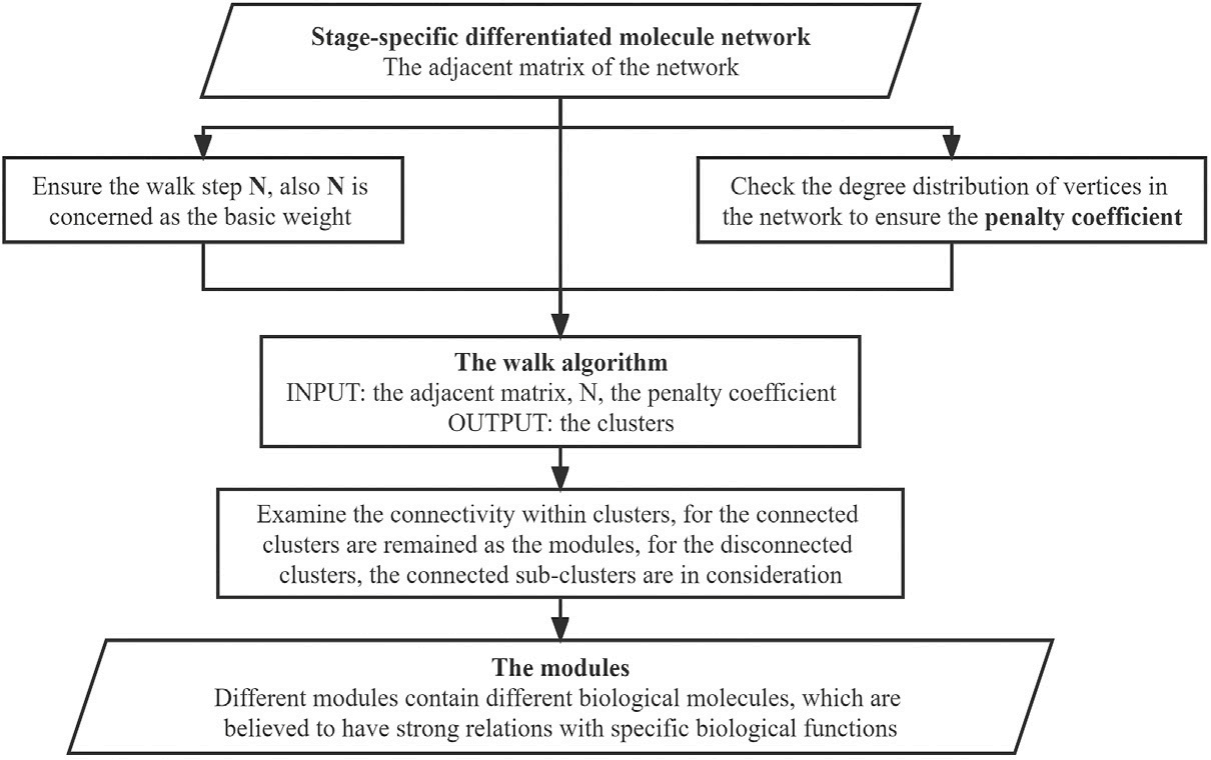
Flowchart for the module identification.

The modules-screening step involved making clusters and screening the connected clusters as modules. We used the union that we obtained in the previous part to generate the clusters, which obeys the rule: if there is an intersection between different unions, the unions should be merged into a vertices list; and the final integrated vertices lists should contain five or more individual vertices in the cluster. Since the vertices in the clusters were screened based on their weights through the walking algorithm, there is one special situation we need to pay attention to. Say there are three vertices A, B, and X. A and B are not directly connected but linked through X. While A and B have high weights and are reserved through the algorithm, X has a low weight and needs to be removed. This will lead to A and B becoming disconnected. Thus, the connectivity within clusters was examined visually using Cytoscape. The connected clusters were directly set as modules, while for the disconnected clusters, the connected sub-clusters were also considered. The specific work in this step is shown in Figure 5.

### 2.4 Building the core networks

In the core-networks-building step, the individual modules obtained from the aforementioned stage-specific networks were taken as vertices to build a global module network, where the connections between modules within the stage were based on the connections of edges between the vertices of the module in the original network, and the connections between modules of two adjacent stages were based on their Jaccard similarity. The reason for building the global module network is to explore the relationship of modules along with the progression of cancers, since the real cancer-related modules tend to form densely connected structures in the global module network, while the non-cancer-related modules tend to be isolated in the network.

After the modules are identified, the biological functions corresponding to individual modules can be obtained through GO and KEGG enrichment analysis. By analyzing the biological functions, we can reveal the relationship of the biological functions with the disease. The *p*-value of the biological functions was acquired through GSEA and then adjusted for multiple tests by using false discovery rate (FDR) (Jafari and Ansari-Pour, 2019; Pike, 2011), and the adjusted *p*-value is the *q*-value. The biological functions with the *q*-value $\leq 5\times 10^{-}2$ were screened as the module-related functions.

Subsequently, the selected biological functions were taken as vertices, and we used the Jaccard coefficient to evaluate their similarity to generate a functional interaction network for further analysis.

The global module network and the functional interaction network both present the statistical information about the disease. Since we obtained the functions in the modules, it can be proven that there is a relationship between the functions and the modules. Therefore, the aforementioned two kinds of networks can be combined to generate a comprehensive network. In this comprehensive network, we combined the two networks with the relationship between the modules and functions as new edges. The comprehensive network combines the two aspects of the disease (biological molecules and biological functions), as well as combining information about the four stages. We thus took both modules and functions as vertices and took three kinds of relationships (between modules, between functions, and between modules and functions) as edges.

In the comprehensive network, to select the core modules, the degree of the modules was taken into consideration. After statistical analysis of the degree of the modules in the network, the distribution of modules with a degree <5 was 64.29%, and this distribution is close to the distribution corresponding to (*μ* − *σ*, *μ* + *σ*) in the normal distribution, which corresponds to 68.26% (Ahsanullah et al., 2014). Therefore, according to the three-sigma rule (Pukelsheim, 1994), we chose modules with degree >=5 to screen out most modules and only retain some typical modules. Therefore, after the statistical analysis, from the comprehensive network, modules with a degree ≥5 were regarded as core modules.

**FIGURE 6:**
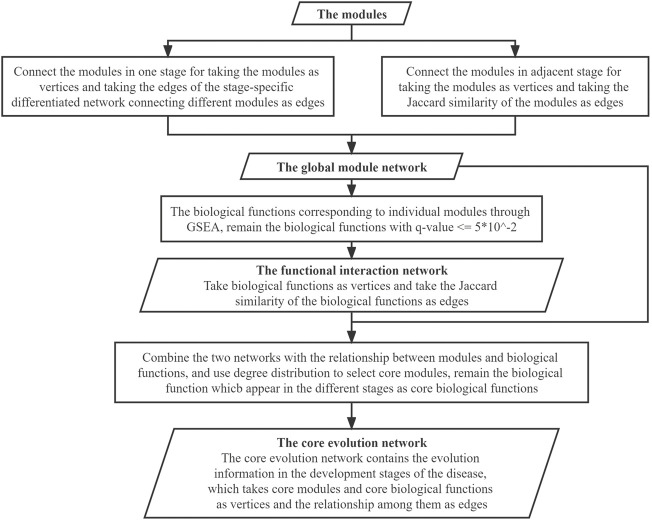
Flowchart for building the core networks.

Ultimately, the core evolution network was generated after vertices selection, which is a subgraph of the comprehensive network. What the core evolution network presents is the dynamic evolution of the disease from the aspects both of biological molecules and biological functions during the four stages of development of the disease. The specific work in this step is shown in Figure 6.

## 3 Results

### 3.1 Core networks

**FIGURE 7:**
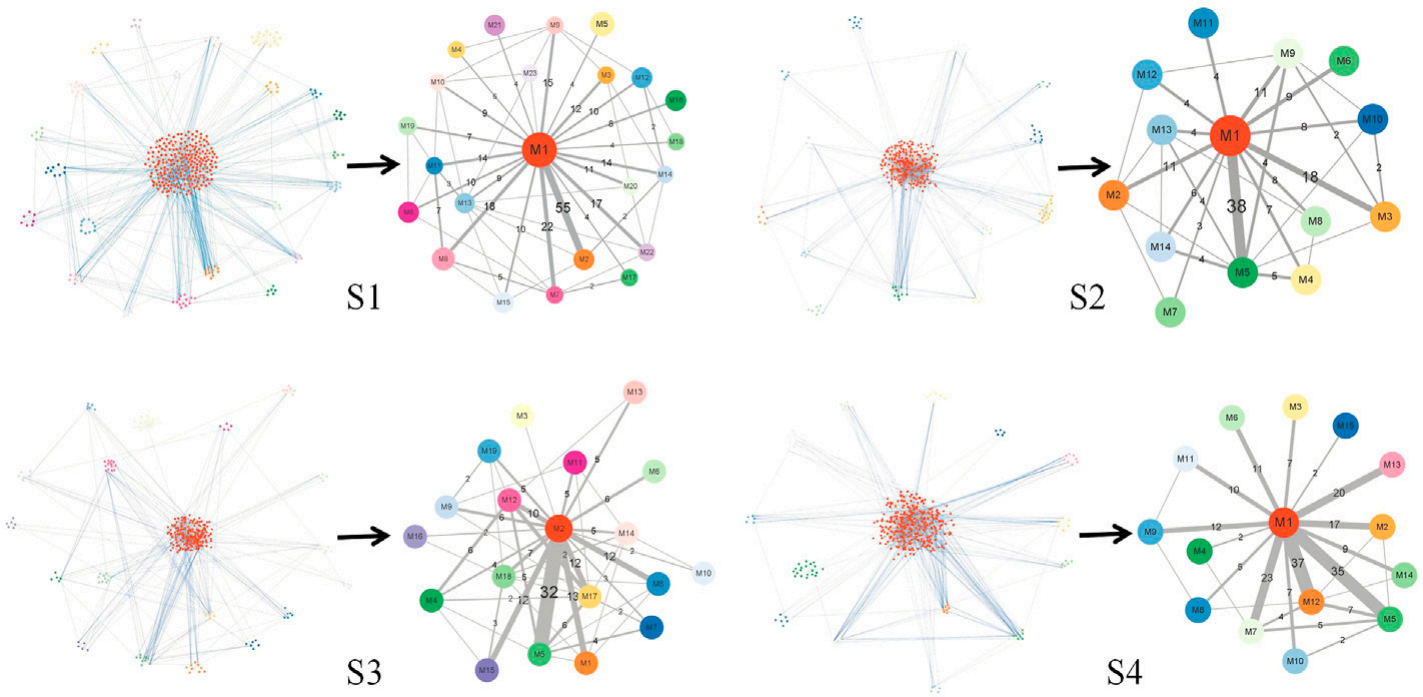
Module network and the simplified module network in *S*1 to *S*4. The figure in each stage has two subfigures: the left one is the module network, with different colors representing biological molecules in different modules, blue edges connecting molecules within different modules, and gray edges connecting different modules; the right one is the simplified module path graph, where the letter *M* on vertices represents module and the number after *M* is the serial number of the module, the number on edges represents the number of edges connecting the two modules, and the width of the edge represents the magnitude of the number.

Figure 7 illustrates the results of module network of *S*1, *S*2, *S*3, and *S*4, respectively. Here, regarding the two kinds of graphs, the left subgraph is the module network in each stage, which takes biological molecules as vertices and the relationship between biological molecules as edges, while the right subgraph is the simplified module network, which takes modules as vertices and the relationship between modules as edges. In the module network, we used different colors of vertices to distinguish different modules and different kinds of edges. Blue edges represent connections between raw molecules, while gray edges represent connections between modules. In the simplified module network, the letter *M* represents the module, and the number after *M* is the serial number of the module. The number on the edge represents the number of edges between biological molecules in the two modules, and the width of the edge represents the magnitude of the number. We used the simplified module network to obtain the main information in the global module network for further analysis.

**FIGURE 8:**
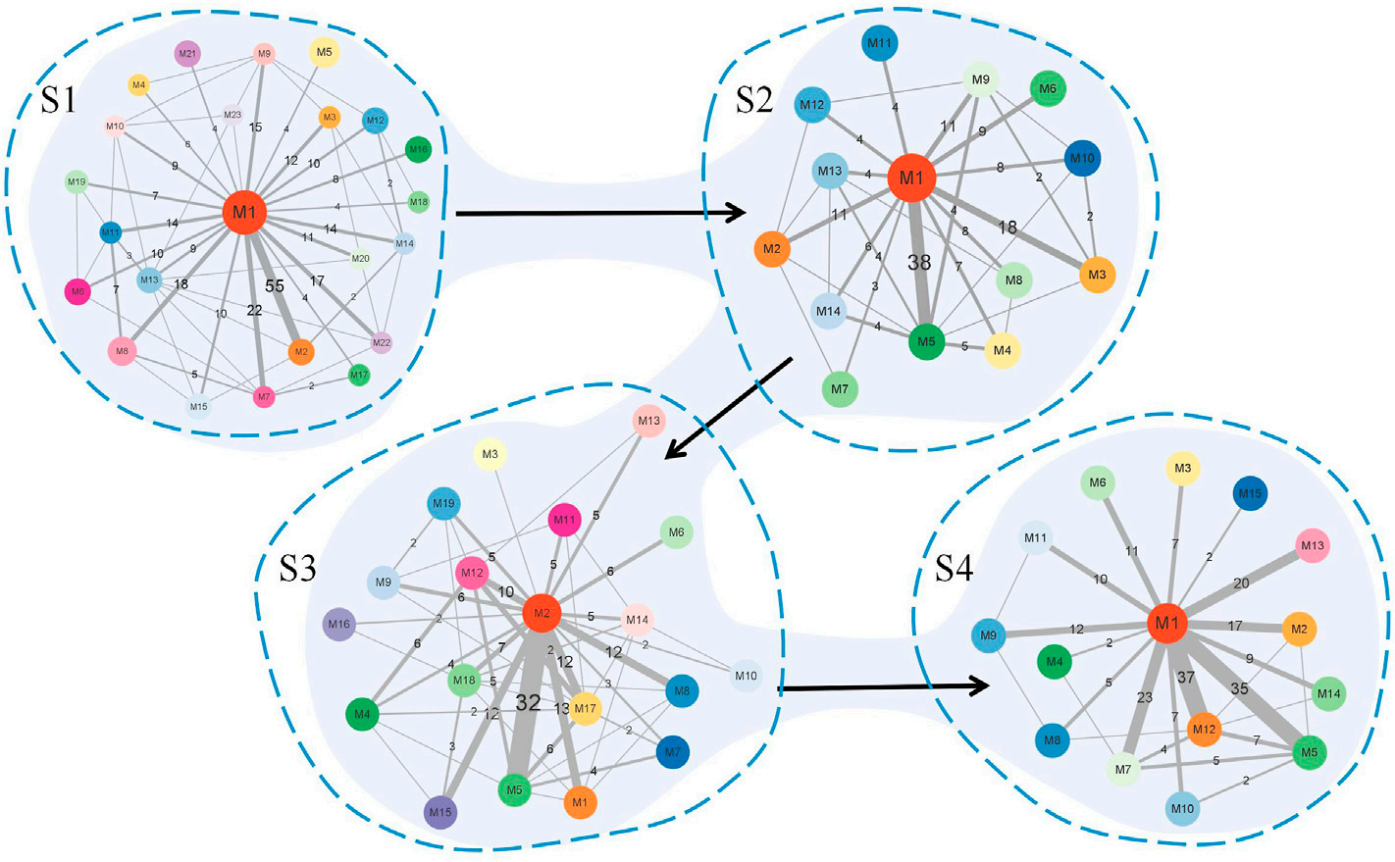
Summary figure of the simplified module networks in the four stages. The figure contains four simplified module networks, each representing a corresponding stage, with arrows marking the progression of the disease according to the order *S*1 → *S*2 → *S*3 → *S*4.

Figure 8 summarizes the simplified module networks among the four sequential stages from *S*1 to *S*4.

**FIGURE 9:**
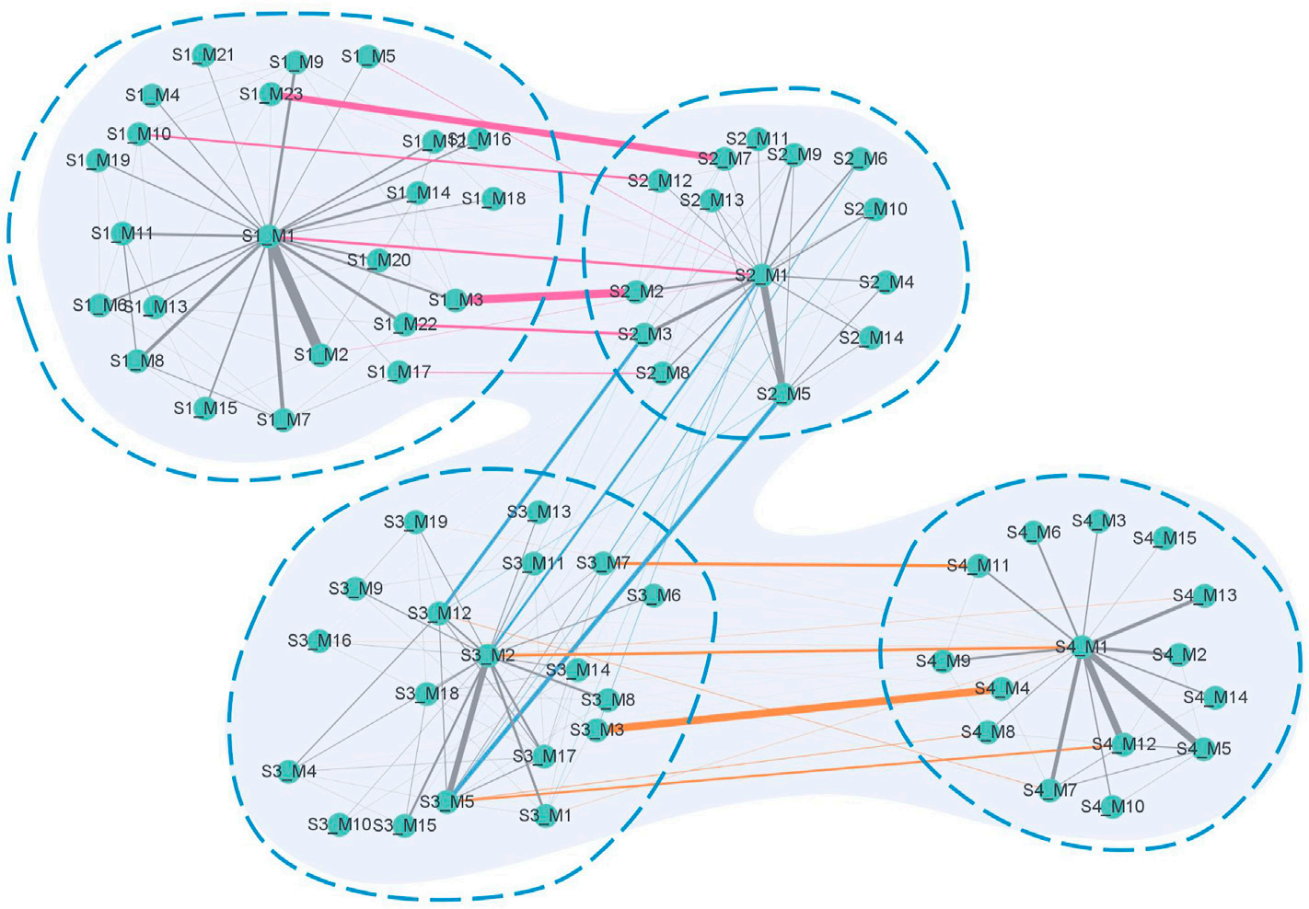
Global module network. The network contains four parts corresponding to the four stages of the disease. Information on the vertices contains a number after the letter *S*, representing the stage serial number, and a number after letter *M*, representing the serial number of the module in each stage. Pink edges connect modules between *S*1 and *S*2, blue edges connect modules between *S*2 and *S*3, and orange edges connect modules between *S*3 and *S*4. The gray edges connect modules in each stage. The width of the edge represents the value of the Jaccard coefficient of the biological molecules in the connected modules.

Figure 9 shows a global module network formed by connecting the simplified module networks in adjacent stages. The pink edges represent the edges connecting *S*1 and *S*2, the blue ones represent the edges connecting *S*2 and *S*3, and the orange ones represent the edges connecting *S*3 and *S*4. The width of the edges represents the value of the Jaccard coefficient of the biological molecules in the connected modules.

**FIGURE 10:**
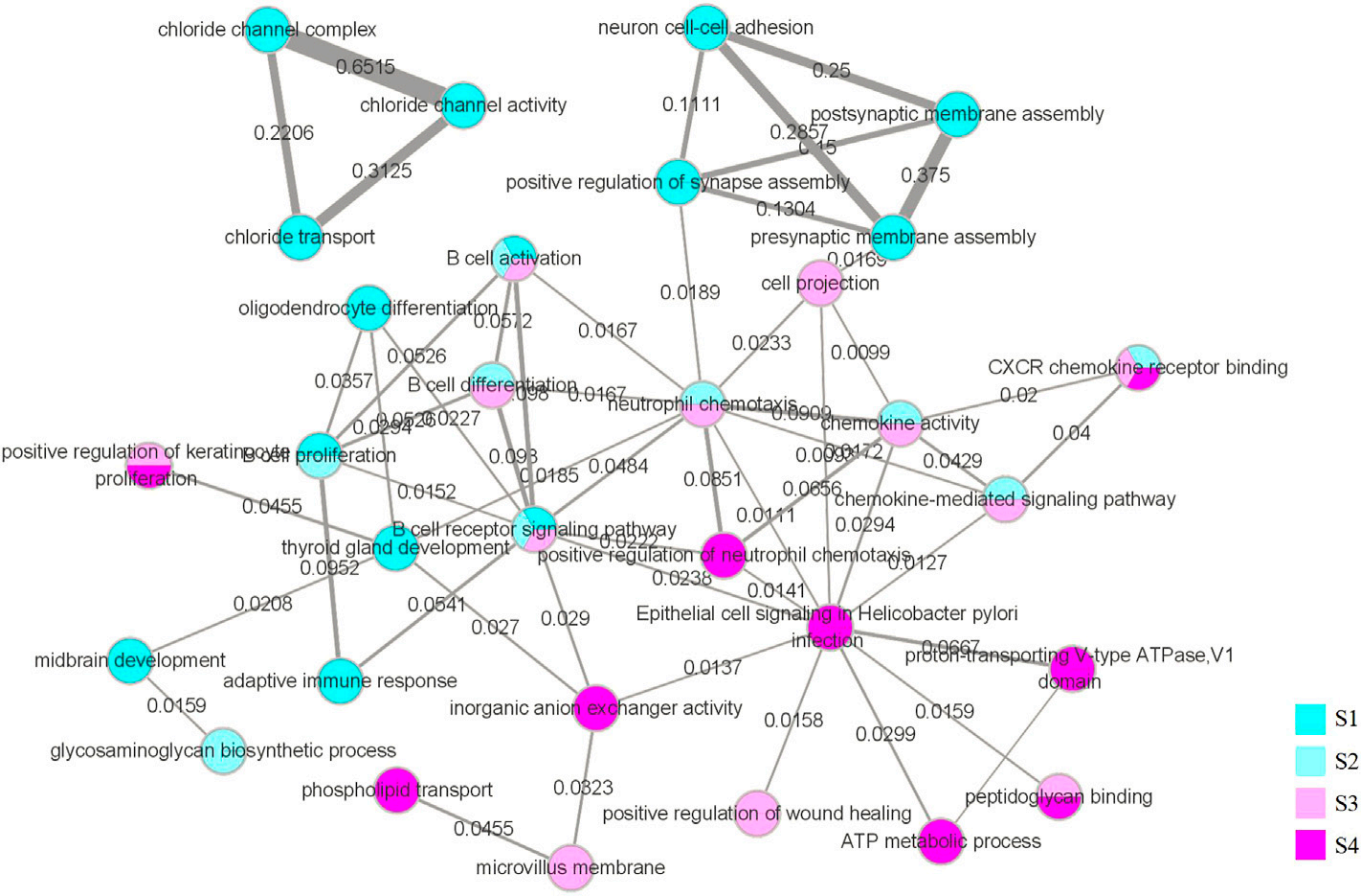
Functional interaction network. The vertices in the network represent the biological functions we obtained through GSEA and selection. The color of the vertices represents the stage in which we obtained the function, and vertices with multiple colors indicate that the corresponding function appears in multiple stages. Using the DAVID database, we obtained all the genes corresponding to the biological functions, and the edges represent that the same genes corresponding to the two biological functions have an intersection. The weights on the edges and the width of the edges represent the value of the Jaccard coefficient of the two groups of genes corresponding to the two biological functions.

While the functional interaction network was generated through GSEA and further screened, we marked the value of the Jaccard coefficient on the edge and used the width of the edge to represent the magnitude of the value. The network is illustrated in Figure 10.

**FIGURE 11:**
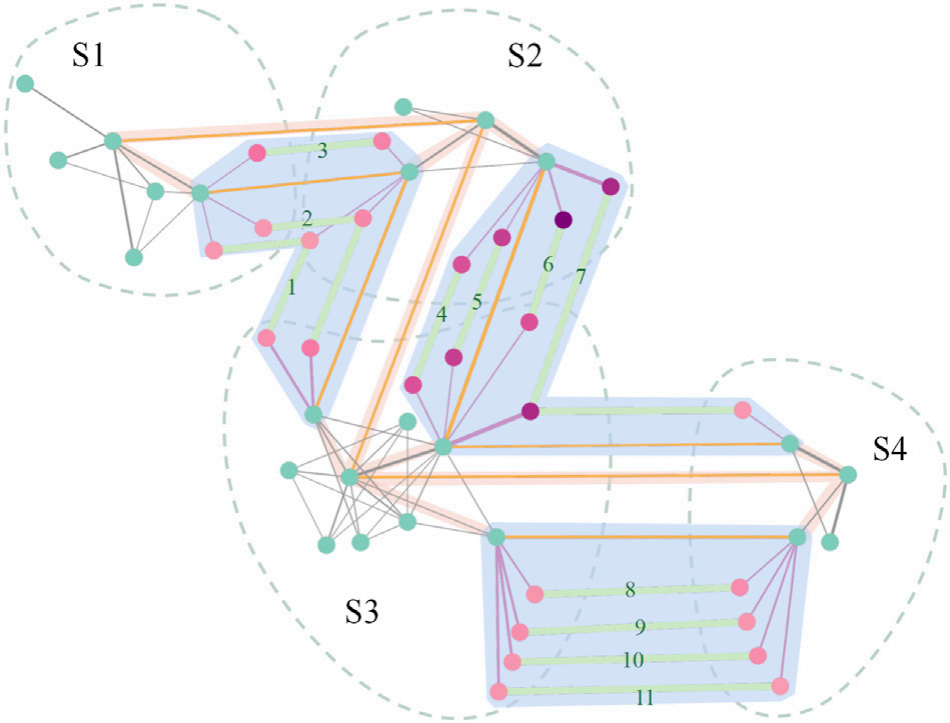
Core evolution network. There are two types of vertices in the network. Green vertices are the core modules, which are the modules combining the global module network and the biological functional network with a degree ≥5 . The pink vertices are the core biological functions that we obtained through GSEA, the selection of which is related to the core modules: the darker the color is, the smaller the *q*-value of the biological functions. The cycles with a dotted line represent the stages of the disease, and the vertices in the cycle represent the core modules and core biological functions in the stage. There are three types of edges. Edges in gray connect the modules in each stage or between the core modules and core biological functions. Edges in orange connect core modules in the two stages with the Jaccard coefficient ≥0.1 . Edges in light green connect the core biological functions, which represent the evolution of the biological functions, and the numbers on the edges represent the serial number of the core biological functions. The blue background highlights the evolution of the biological functions and the corresponding 11 core biological functions, which are as follows: 1. B-cell differentiation; 2. B-cell activation; 3. B-cell receptor signaling pathway; 4. neutrophil chemotaxis; 5. chemokine activity; 6. chemokine-mediated signaling pathway; 7. CXCR chemokine receptor binding; 8. peptidoglycan binding; 9. negative regulation of keratinocyte differentiation; 10. oligosaccharide binding; and 11. positive regulation of keratinocyte proliferation.

After combining the global module network and functional interaction network with edges between modules and biological functions to form the comprehensive network, the core modules and core biological functions were selected to construct the core evolution network. This network is shown in Figure 11. We used green vertices to represent modules, and pink vertices to represent functions with different shades. The smaller the *q*-value of the function, the darker the color. Furthermore, the yellowish pink background represents the modules that we show in detail, and the number represents the sequence of the functions. The result obtained prove that the biological functions associated with the B cell and CXCR family play significant roles in LUAD (Gangadhar et al., 2010; Wang et al., 2019; Liao et al., 2021; Hu et al., 2022). Therefore, among the core biological functions we obtained, the biological functions of B-cell differentiation, B-cell activation, B-cell receptor signaling pathway, and CXCR chemokine receptor binding are associated with LUAD. The results also demonstrate that chemokine-related biological functions are applied in clinical treatments (Tsukita et al., 2019; Liu and Wu, 2021); thus, the biological functions of chemokine activity and the chemokine-mediated signaling pathway can provide certain evidence for the diagnosis of the disease. It is worth noting that the LUAD-related biological functions of the B cell and the CXCR family are in a relatively broad range; the study narrows the broad concept to the four specific biological functions and reflects their roles in the evolution process of the disease more accurately. However, the biological functions of the negative regulation of keratinocyte differentiation and the positive regulation of keratinocyte proliferation normally do not show in LUAD (Xu et al., 2020). The result is only on the statistical analysis level. Similar to the biological functions of peptidoglycan binding and oligosaccharide binding, the four biological functions were obtained through GSEA with the two key RNAs in the core modules: REG3A and REG3G (see Figure 13). Therefore, due to the limitations of GSEA (Tamayo et al., 2016), the calculation results are only based on statistical analysis, and they still need to be verified and screened in combination with more biological evidence. Furthermore, the biological functions of neutrophil chemotaxis, peptidoglycan binding, and oligosaccharide binding are associated with some diseases, but there is no direct evidence that they are strongly associated with LUAD (Metzemaekers et al., 2020; Cui et al., 2022; Rye and Bovin, 1997). Thus, the biological function of neutrophil chemotaxis can be used as a prediction for clinical pretreatment, and the biological functions of peptidoglycan binding and oligosaccharide binding may serve as a predictive signal that requires more biological evidence. In summary, of the 11 biological functions we obtained, six are related to the disease, the biological function of neutrophil chemotaxis is not directly associated with LUAD but can serve as a predictor, two functions may serve as a predictive signal, and two functions need to be verified through more biological evidence.

Since few biological functions are performed by isolated biological molecules, we need to find the common participation of multiple biological molecules corresponding to biological functions. That is why we first divided the biological molecules into different modules in the previous steps and divided the closely related biological molecules into different modules. Furthermore, we used the biological molecules inside the module to find the corresponding biological functions. The core biological functions and the corresponding biological molecules are shown in detail in Table 1.

**TABLE 1:** Biological molecules corresponding to the core biological functions.

Core biological function	Biological molecules
B-cell differentiation	CR2, CD79A, CD79B, CD22, MS4A1
B-cell activation	CR2, CD79A, CD79B, CD22, MS4A1, PRKCB
B-cell receptor signaling pathway	CR2, CD79A, CD79B, CD22, PRKCB
Neutrophil chemotaxis	SAA1, END2, END3, CCL13, CCL8, CCL26
	CCL23, CXCL11, CXCL8, CXCL5, CXCL2, CXCL1
	PPBP, CXCL3, CXCL6, PF4, CCL19, TREM1, CCL14
Chemokine activity	CXCL1, CXCL2, CXCL5, CXCL6, CXCL8, CCL19
	PF4, CXCL3, PPBP, CCL13, CCL26, CXCL11
	CXCL12, CCL14, CCL28, CCL8, CCL23
Chemokine-mediated signaling pathway	CXCL5, CXCL2, CXCL8, CXCL1, CXCL11, CCL26
	PF4, CCL14, CCR2, CXCL6, CXCL12, CCL13
	PPBP, CCL23, TFF2, CCL8, CCL19, CXCL3
CXCR chemokine receptor binding	CXCL1, CXCL2, CXCL5, CXCL8
Peptidoglycan binding	REG3G, REG3A, ZG16
Negative regulation of keratinocyte differentiation	REG3G, REG3A, MSX2

Oligosaccharide binding	REG3G, REG3A, ITLN1
Positive regulation of keratinocyte	REG3G, REG3A, AREG, CDH3
Proliferation	

**FIGURE 12:**
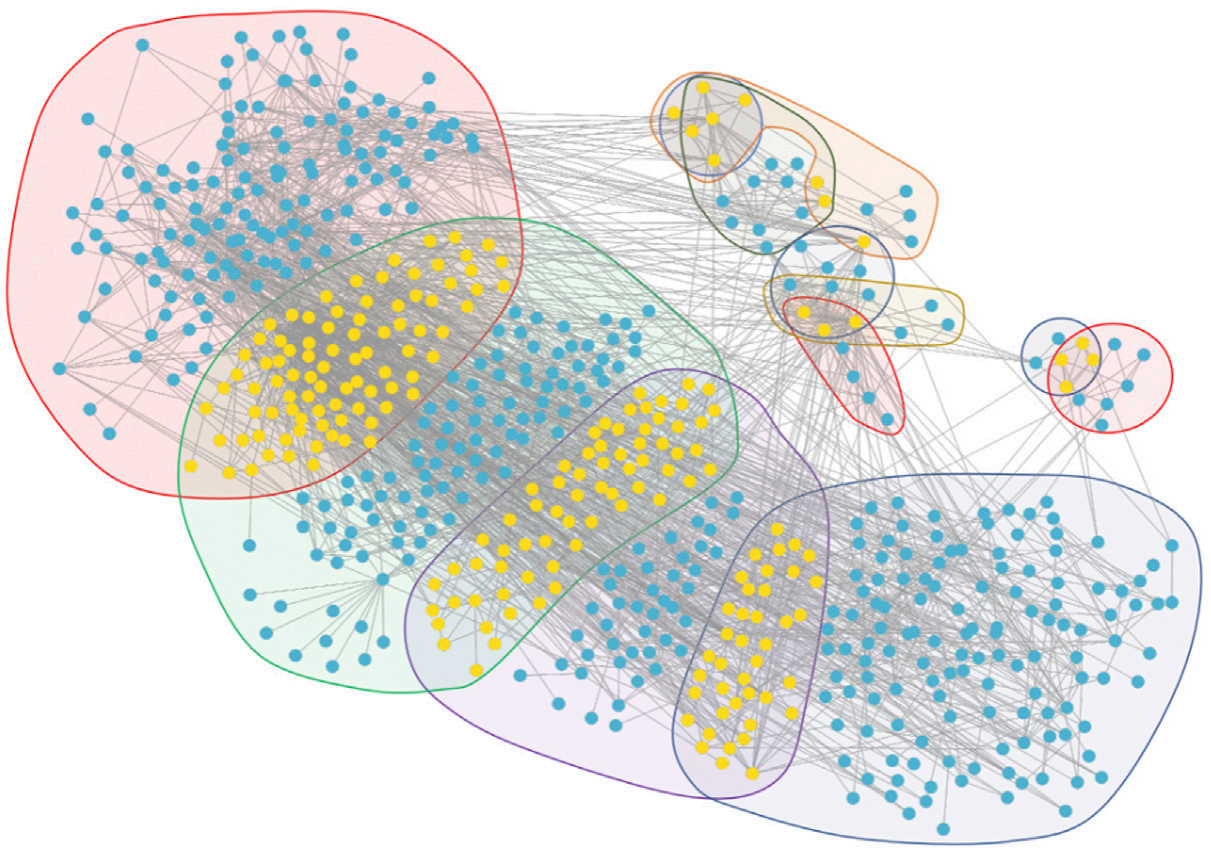
Module overlap visualization. The vertices in the visualization contain yellow vertices and blue vertices. The yellow vertices are the intersection of biological molecules of the modules, while the blue vertices are not the intersection. The background regions in different colors represent different modules. The four large regions represent the four largest core modules, corresponding to the four stages which, through GSEA, do not obtain accurate results. The other eight small regions represent the eight core modules which, through GSEA and selection, reveal the core biological functions.

From the core evolution network, we selected 12 core modules and 11 core biological functions, and the details are visualized in Figure 12. In this figure, the biological molecule corresponding to the vertex lies in different regions with different background colors. If the biological molecule appears in multiple modules, the biological molecule lies in the intersection corresponding to the overlapping region of the modules.

**FIGURE 13:**
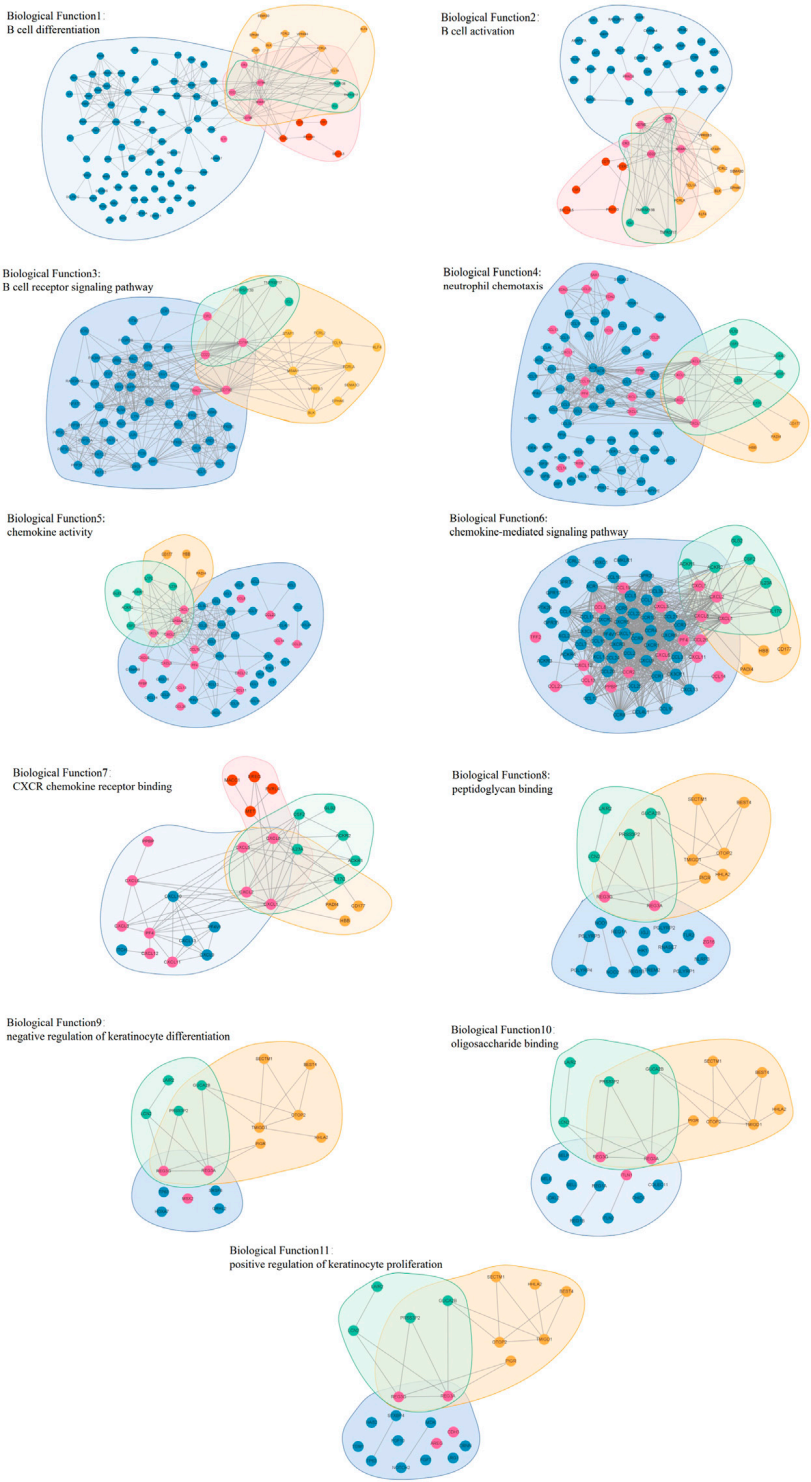
Graphs (11) visualizing the biological functions overlap.

Figure 13 shows the relationship between overlapping core biological functions. The biological molecule dataset related to different biological functions was first obtained from the DAVID database, and the connections between these molecules were obtained from the STRING database. The blue background represents the biological molecules related to the biological functions, and the yellow, green, and red background represent the biological molecules in different modules. Vertices not in blue are the biological molecules in the original network, while vertices in blue mean otherwise.

### 3.2 Comparison

Regarding comparison, we primarily designed two control groups called Design1 and Design2. For Design1, we directly put all the biological molecules in each stage into GSEA to obtain the results. For Design2, we put the biological molecules obtained through our clustering algorithm, without dividing them, into different modules into GSEA to obtain the results.

We compared these two design methods to our method to find the different stages of the 11 biological functions according to the stage separation to compare the *q*-values of the 25 biological functions. These 25 items were generated into a heat matrix. Moreover, we combined a hierarchical clustering algorithm to cluster the information corresponding to these 25 biological functions. The specific clustering effect is shown in the left part of Figure 11, which is helpful for further exploration and analysis.

**FIGURE 14:**
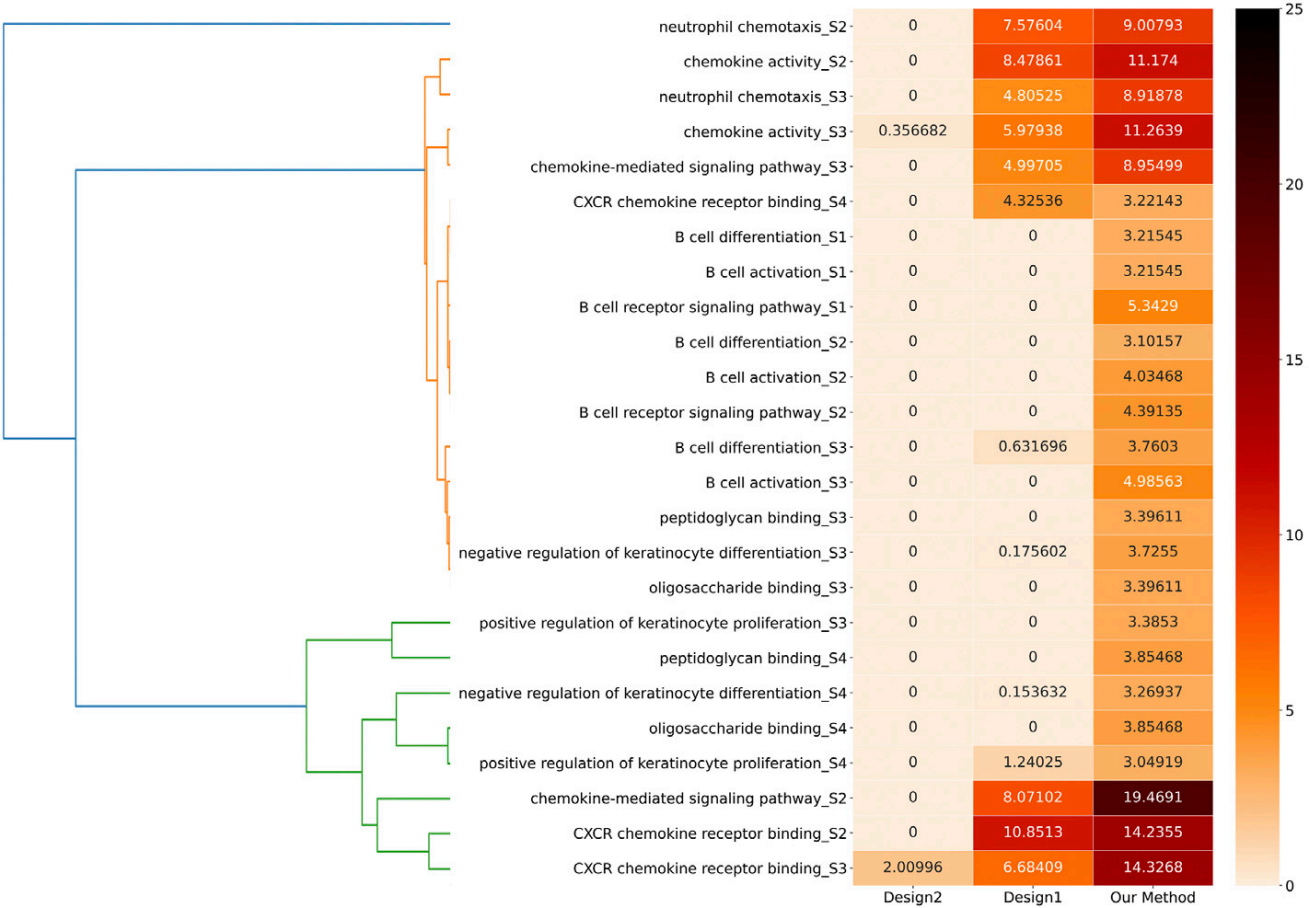
Effectiveness comparison of the three methods. In the heat matrix, we take *Q*′ = − ln *q* as the comparative index and some biological functions cannot be found in the corresponding enrichment information in some methods. We use 0 to represent absence or a *q*-value equal to 1.0; thus, the larger the value of *Q*′, the smaller the *q*-value and the darker the color.

Figure 14 shows the comparisons among these three methods. On the one hand, our method demonstrates significance for the majority of biological functions, while Design1 and Design2 in many biological functions lack corresponding significance according to their *q*-values. On the other hand, the *q*-value of our method is smaller than that of Design1 and Design2 in all cases. Therefore, our method can reveal these biological functions more accurately and effectively. Our result is better than that of Design1 (which shows that it is better to cluster the whole network) and also better than that of Design2 (which shows that it is better to build modules than to put vertices together).

## 4 Conclusion

In this study, we have proposed a method to investigate the evolution of LUAD from the perspective of the combination of graph theory knowledge and biological knowledge. The global module network, the functional interaction network, and the core evolution network were presented from the dynamic evolution point of view. In the core evolution network, there are 12 core modules and 11 core biological functions. Among the core biological functions, six are related to the disease, the biological function of neutrophil chemotaxis is not straightly associated with LUAD but can serve as a prediction, two functions may serve as a predictive signal, and two functions need to be verified through more biological evidence. The core evolution network highlights the evolution information for the multi-stage disease.

The advantages of the proposed method are as follows. First, we chose an innovative clustering algorithm based on the random walk algorithm and the Monte Carlo method for the network clustering and designed a set of parameters for each step of the experiment to find the best parameter setting based on statistical principles. Additionally, many objective factors were considered in each step of the algorithm design, and some simple ideas or models were used to solve problems. Second, compared with other methods, our method not only presented the information of each stage statically, but also presented the evolution dynamically. Third, the idea of “focusing on the main factors” is implied in every step, which greatly reduced the amount of work and harvested the most typical results.

The limitations of the proposed method are as follows. The algorithm can be further optimized to overcome the high time complexity of the method. Moreover, in the cluster-screening step, we directly combined the union as the clustering results, without considering the corresponding problems of irrelevant vertices, which should be further investigated.

## Data Availability

Publicly available datasets were analyzed in this study. This data can be found here: https://portal.gdc.cancer.gov/, https://www.gencodegenes.org/.
